# The characteristic direction: a geometrical approach to identify differentially expressed genes

**DOI:** 10.1186/1471-2105-15-79

**Published:** 2014-03-21

**Authors:** Neil R Clark, Kevin S Hu, Axel S Feldmann, Yan Kou, Edward Y Chen, Qiaonan Duan, Avi Ma’ayan

**Affiliations:** 1 Department of Pharmacology and Systems Therapeutics, Systems Biology Center New York (SBCNY), Icahn School of Medicine at Mount Sinai School, New York, NY 10029, USA

## Abstract

**Background:**

Identifying differentially expressed genes (DEG) is a fundamental step in studies that perform genome wide expression profiling. Typically, DEG are identified by univariate approaches such as Significance Analysis of Microarrays (SAM) or Linear Models for Microarray Data (LIMMA) for processing cDNA microarrays, and differential gene expression analysis based on the negative binomial distribution (DESeq) or Empirical analysis of Digital Gene Expression data in R (edgeR) for RNA-seq profiling.

**Results:**

Here we present a new geometrical multivariate approach to identify DEG called the Characteristic Direction. We demonstrate that the Characteristic Direction method is significantly more sensitive than existing methods for identifying DEG in the context of transcription factor (TF) and drug perturbation responses over a large number of microarray experiments. We also benchmarked the Characteristic Direction method using synthetic data, as well as RNA-Seq data. A large collection of microarray expression data from TF perturbations (73 experiments) and drug perturbations (130 experiments) extracted from the Gene Expression Omnibus (GEO), as well as an RNA-Seq study that profiled genome-wide gene expression and STAT3 DNA binding in two subtypes of diffuse large B-cell Lymphoma, were used for benchmarking the method using real data. ChIP-Seq data identifying DNA binding sites of the perturbed TFs, as well as known drug targets of the perturbing drugs, were used as prior knowledge silver-standard for validation. In all cases the Characteristic Direction DEG calling method outperformed other methods. We find that when drugs are applied to cells in various contexts, the proteins that interact with the drug-targets are differentially expressed and more of the corresponding genes are discovered by the Characteristic Direction method. In addition, we show that the Characteristic Direction conceptualization can be used to perform improved gene set enrichment analyses when compared with the gene-set enrichment analysis (GSEA) and the hypergeometric test.

**Conclusions:**

The application of the Characteristic Direction method may shed new light on relevant biological mechanisms that would have remained undiscovered by the current state-of-the-art DEG methods. The method is freely accessible via various open source code implementations using four popular programming languages: R, Python, MATLAB and Mathematica, all available at: http://www.maayanlab.net/CD.

## Background

Genome-wide transcriptional profiling, the parallel measurement of the expression of tens of thousands of genes, is a powerful tool which, for example, aids in the development of clinical biomarkers for disease diagnosis, reveals the heterogeneity of histologically identical cancers, and sheds light on diverse biological mechanisms. After estimating the relative or absolute expression level of all transcripts, the next step is to test statistical hypotheses [1]. Typically, these hypotheses are concerned with the difference between two biological conditions, for example, normal verses diseased tissue, or perturbed verses unperturbed cells. One of the most important aims of such tests is to identify the genes which are mostly responsible for the difference between the biological states under investigation, the so called differentially expressed genes (DEG).

**Figure 1:**
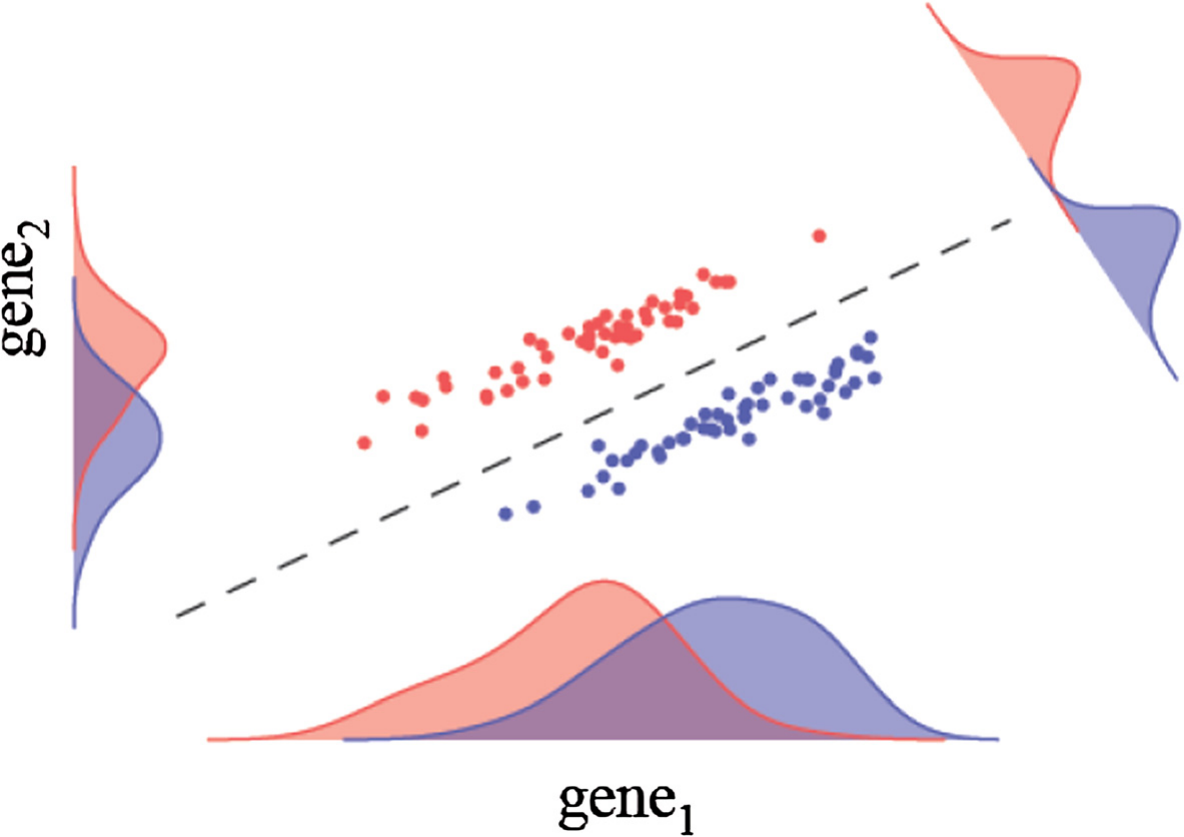
**Illustration of a case where there is no marginal differential expression of individual genes, however in the multivariate setting the differential expression becomes clear.** Projecting the data onto the appropriate direction in this case leads to a clear separation between the classes.

Genes do not function in isolation but are part of a complex regulatory and functional network, and this can be reflected in the significant observed correlations between their expression levels. However, the most widely used methods for identifying DEG are univariate; typically tests are performed gene-by-gene without regarding gene-gene statistical dependencies. The fold-change, an early approach that is not recommended by statisticians but still popular among experimental biologists due to its simplicity, does not take into account the variance which arises from biological and experimental sources, and as such the fold-change measure does not offer any estimate of confidence [2,3]. Because of this, the fold-change is regarded as an insufficient statistic for identifying DEG [3,4]. Other univariate methods include, Welsh’s t test, Significance Analysis of Microarrays (SAM) [5], and Linear Models for Microarray Data (limma) [6], and, in the case of high-throughput sequencing data, differential gene expression analysis based on the negative binomial distribution (DESeq2) [7]. However, since there are significant statistical dependencies between the expression levels of most genes, multivariate approaches may be more appropriate for genome-wide profiling analyses that identify DEG; for example, multivariate analysis is able to find significant differential expression in cases where there is no marginal differential expression for individual genes (Figure 1).

There have been a number of attempts to apply multivariate analyses to identify DEG [8,9]. For example, Lu et al. [10] proposed an application of Hotellings *T*^2^ test, which is a multivariate generalization of Welsh’s t-test. However, these approaches remain little-used because they are sensitive to the fact that typically microarray or RNA-Seq gene expression profiles have fewer samples than genes. A small sample size compared to the dimensionality of the measured variables brings difficulties to the analysis [11]. A significant step towards the resolution of such problems was the realization that variance shrinkage improves statistical power [5,12,13]. Also, methods that directly attempt to identify differentially expressed gene-sets as opposed to individual genes have been developed [14-19]. In addition, to increase statistical power, these approaches also attempt to facilitate biological interpretation, which can be challenging when faced with a long list of DEG [15,20].

There are currently two main principle technologies to perform whole-genome transcriptional profiling: microarrays and RNA-Seq. The later has a number of advantages such as greater dynamic range, and an ability to measure previously unknown transcripts. The RNA-Seq technology also presents some challenges such as potential non-uniform read coverage and transcript length biases, and recently there has been a flurry of publications approaching these important issues [21-24]. One of the differences between microarray and RNA-Seq data is that microarrays result in continuous measures of expression, often log-normally distributed, whereas RNA-Seq data results in positive integer read counts with discrete probability distributions. For this reason, established methods of differential expression analysis for microarray data are not immediately transferable to RNA-Seq data but this challenge can be overcome as demonstrated by Soneson et al. [25] who showed how to transform RNA-Seq counts to continuous values. The approach we shall take here relies on minimal assumptions about the distributions of the data and should apply to RNA-Seq, microarray or any other similar situation where the dimensionality of the data far exceeds the sample size.

We propose a new multivariate approach called the Characteristic Direction which is better able to identify DEG than univariate approaches including the methods: fold change, SAM, the Welch’s t test, LIMMA and DESeq. Our approach naturally incorporates a regularization scheme to deal with the problem of dimensionality, and also provides an intuitive geometrical picture of differential expression in terms of a single direction. We show how this geometrical picture reliably characterizes the differential expression and also leads to some natural extensions of the approach such as improved gene-set enrichment analysis. In addition, we take advantage of a neat mathematical trick to make the Characteristic Direction method fast to compute.

**Figure 2:**
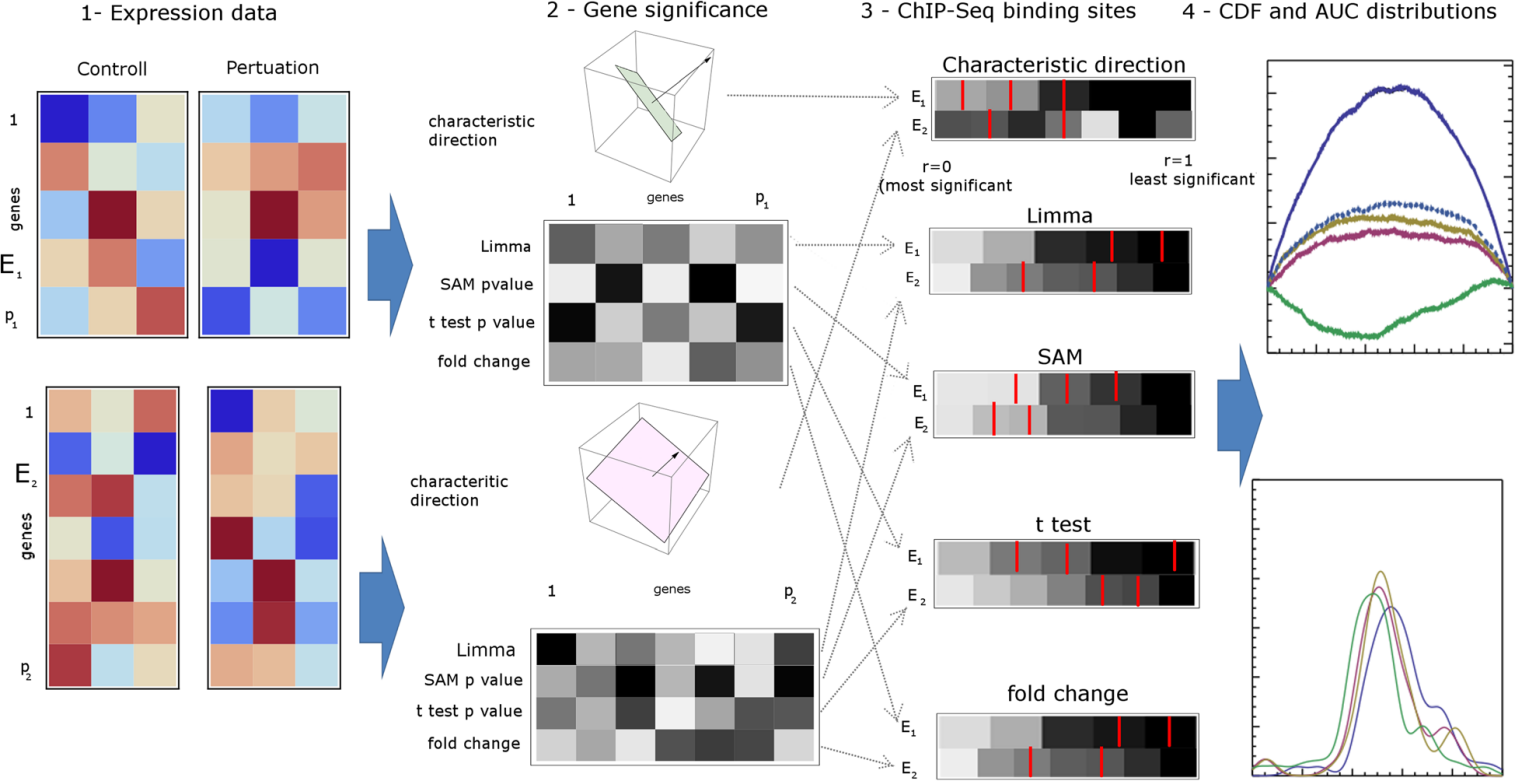
**Schematic of the validation pipeline: 1) Expression data from a large number of experiments with control vs. perturbation samples; 2) The various approaches to differential expression are used to rank genes in order of significance; 3) Prior knowledge gene lists, for example genes associated with ChIP-Seq binding sites of the perturbed TF, are identified in the ranked list and the cumulative distribution is calculated; 4) The perturbation of the cumulative distribution from uniform is examined.** Large deviations from zero, on the scale of φ, indicate significant prioritization of the prior knowledge genes. Also, the AUC distributions are examined across the various methods.

Previous attempts to validate expression analyses have tended to rely on simulated data due to the general lack of ground truth with which to compare the results when applying the methods to real data. However, simulated data can only contain a simplified reproduction of the rich structure of expression data and so can only provide an incomplete picture of the effectiveness of the method under investigation. We benchmarked our method using simulated data, but we also developed original methods to benchmark DEG calling using real data (Figure 2). Firstly, we extracted a large number (73) of gene expression microarray profiles from the Gene Expression Omnibus (GEO) before and after the perturbation of individual TF. We then used ChIP-Seq data, which provides information on the DNA binding sites of the TFs to provide prior information by which we were able to evaluate the Characteristic Direction method and make comparisons to other approaches. We are aware that target genes for a TF, as determined by ChIP-Seq, does not necessarily mean that the binding is functional. However, identifying more DEG from a list of putative target genes determined by ChIP-Seq can be used as a silver standard to compare DEG calling methods. In a similar way, we were also able to do this for drug perturbations (130) in combination with knowledge about the drug targets and their known protein-protein interactions. For validation and evaluation of the Characteristic Direction method ability to identify DEG from RNA-Seq data, there are currently fewer available studies from including set up similar test-beds, mostly because the technology is newer and cost prevents many studies from including more than two repeats. However, we did find an ideal study that measured binding sites for the TF STAT3 and relevant RNA-Seq expression data in the same cells.

Apart from being able to assess the analysis methods, these results are interesting in themselves as they show that we are able to infer information about TF and drug perturbations from expression data. For example, we find that proteins that are known to interact with the TFs and the drug targets tend to be also within the DEG. Finally, we show how a natural extension of the Characteristic Direction method can be used to perform potentially improved gene-set enrichment analysis. We compared enriched terms for DEG identified in human cancer stem cells and show that the Characteristic Direction enrichment method recovers more relevant Gene Ontology (GO) terms as compared with GO terms recovered by the hypergeometric test, or GSEA.

## Methods

### Computing the characteristic direction and identifying differentially expressed genes

Classification approaches, for example those that predict clinical outcome from gene expression data, are inherently multivariate as they use the structure of the gene expression profiles as a whole in order to distinguish between biological conditions or classes. Our approach is to repurpose linear classification methods in order to characterize differential expression and identify DEG. We use a linear classification scheme, which defines a separating hyper-plane; the orientation of which we show can be interpreted to identify DEG. We also find that the direction normal to the separating hyper-plane provides a simple geometrical conceptualization of the differential expression, which naturally leads to extensions of the approach, such as a new formulation of gene set enrichment analysis.

Suppose we have gene expression data from a number of samples *N*, in which the expression of *p* genes is measured, and then let each expression profile sample form a row of the matrix ***X*** (a *N* × *p* matrix). For generality at this point we shall consider the case where each of the expression samples comes from one of *K* classes belonging to the set *G*. In linear discriminant analysis (LDA) the log-ratio of class posteriors *P* (*G*|*X*), is written as follows (see Additional file 1 for a derivation),

(1) $$\begin{array}{l} \log\frac{\mathrm{Pr}\left(G=k|X=x\right)}{\mathrm{Pr}\left(G=l|X=x\right)}=\log\frac{\pi_{k}}{\pi_{l}}-\frac{1}{2}{\left(\mu_{k}-\mu_{l}\right)}^{T}\Sigma^{-1}\left(\mu_{k}-\mu_{l}\right)\\ \;+x^{T}\Sigma^{-1}\left(\mu_{k}-\mu_{l}\right) \end{array}$$

where, *π*_*k*_, is the class mean, and it is assumed that both classes have the same covariance matrix, Σ. Then the orientation of the separating hyper-plane (between classes *k* and *l*) is defined by the normal *p*-vector, in the third term on the right hand side, that we label *b*,

(2) $$b=\Sigma^{-1}\left(\mu_{k}-\mu_{l}\right).$$

The estimation, from the data, of the terms in this equation is explained in the Additional file 1. Below we will interpret the direction of the *p*-vector, *b*, as the direction in expression space that best characterizes the differential expression, and show how the components of this vector can be used to identify differentially expressed genes. However, first we note a few potential issues: the calculation involves the inverse of a very large *p* × *p* matrix which is not only expensive to compute but also the elements must be estimated from a relatively small sample-size (*p* >>*N*), which means that the matrix is singular and this leads to large variance in the results even when using the generalized inverse.

The issue of singularity and large variance can be tackled with a regularization procedure, for example, the covariance matrix can be shrunk to the scalar variance as follows,

(3) $$\hat{\Sigma }\left(\gamma \right)=\gamma \hat{\Sigma }+\left(1-\gamma \right)\sigma^{2}I_{p},\mathrm{with}\;\gamma \epsilon \left[0,1\right]$$

where $\hat{\Sigma }$ is the estimated covariance matrix, and *σ*^2^ is the scalar covariance (see Additional file 1 for elaboration). The inclusion of a constant on the diagonal resolves the singularity problem, and the modulation of the off-diagonal terms helps to reduce noise arising from the estimation of covariance from few samples.

The problem of computational expense is efficiently overcome with the singular-value decomposition trick [26-28] which also admits a solution in the limit of zero shrinkage by working in the subspace spanned by the data, rather than the full expression space (see the Additional file 1). The normalized vector $\hat{b}$ contains only information about the direction of the normal to the separating hyper-plane. The components of $\hat{b}$ are the direction cosines, and their magnitude quantifies the degree of alignment of the direction to axes corresponding to each gene. The sign of each component can be interpreted as the sign of the contribution of each gene to the differential expression. Another way to picture this interpretation of gene significance is to consider the identity,

(4) $$\sum_{i=1}^{p}{\hat{b}}_{i}^{2}\equiv 1$$

Then the contribution of each ${\hat{b}}_{1}^{2}$ to this sum can be interpreted as quantifying the relative contribution of each component to the total differential expression giving the significance of the corresponding gene. The above interpretation provides a quantitative measure of the relative, but not absolute, significance of each gene to the differential expression, and as such can be used to rank the genes in order of significance. However, we also want to identify a shortlist of significant DEGs. This could be done completely within the framework we have outlined by using a *L*_1_ regularization scheme in place of that used in the shrinkage equation above; such a penalty results in automatic feature selection because many components fall to zero; the genes corresponding to the features retained would then comprise the DEGs. An alternative method to deriving a significance threshold is described below.

### Generating synthetic data

We generate synthetic normalized expression data which incorporates multivariate structure. The multivariate structure of real biological expression data is not fully known, we therefore use a simple approach which incorporates some of the best established properties of such data: 1) large number of features (genes) with a relatively small number of samples; 2) significant dependencies between the expression levels of the genes, leading to dimensionality which is much smaller than the number of features. In addition to these properties we require control over the number and identity of genes which are differentially expressed between two datasets. There are a number of ways that datasets with these properties may be generated, but we chose the simplest, with the fewest free parameters. In a nutshell, we use a multi-variate normal distribution distributed throughout a random subspace of the full expression space, the dimension of which reflects the dimension of the dataset. By ensuring that this subspace spans a predefined vector of differentially expressed genes we can perturb the mean of the normal distribution, preserving the covariance matrix, to generate data with pre-defined differentially expressed genes. An explicit description of the algorithm follows:

The parameters input into the synthetic data generation algorithm are: the total number of genes on the array, *p*, the total number of differentially expressed genes, *n*_*d*_, the dimension of the data sets, *D*, the number of samples in each class, *N*, and a scale parameter which controls the magnitude of the difference between the “control” and “perturbed” data sets, Δ. First we determine which genes are to be differentially expressed and in which direction – this is done by generating a random unit *p*-vector with *n*_*d*_ non-zero components, corresponding to the differentially expressed genes. We refer to this vector as $\hat{m}$. This vector, when normalized, provides the seed for the generation of a set of *D* isotropic random orthonormal vectors which provide a basis for a random subspace of expression space. This is generated by iteratively generating a random isotopic vector *b*_*i*_ at step *i*, then calculating that part of *b*_*i*_ which is parallel to the subspace spanned by the previously generated vectors {*b*_*j*_ |*j* <*i*},

(5) $$b_{i,∥}=\sum_{j=1}^{i-1}\left(b_{i}.b_{j}\right)b_{j}$$

**Figure 3:**
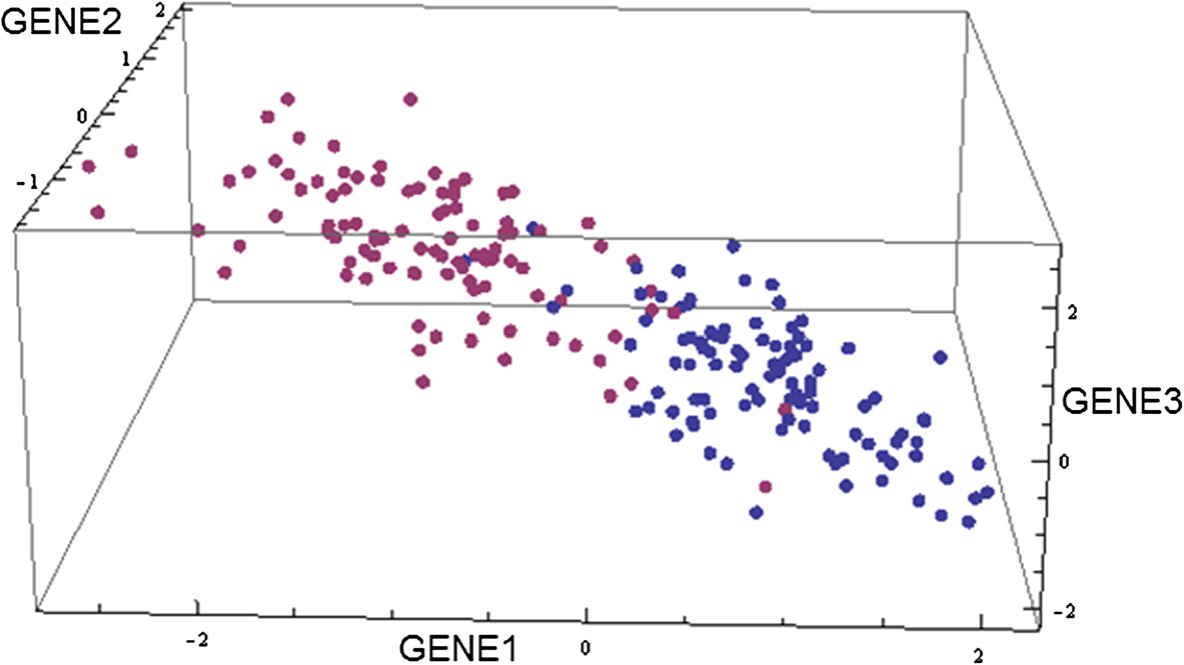
**Illustration of the structure of the synthetic data with parameters: *p *= 3, *n***_***d***_**= 2, *D *= 2, *N *= 3, and Δ = 3.0.** The differentially expressed genes are gene1 and gene3. The two different colors of points indicate the two classes of samples: “control” and “perturbed”.

This is then subtracted from *b*_*i*_, resulting in a new vector which is perpendicular to the previous members of the set; this is normalized before being included in the set and moving on to the next iteration. The result is a set of orthonormal basis vectors for an isotropic subspace of dimension *D* which also includes the pre-defined vector of differentially expressed genes. For each class: “control” and “perturbed” we next generate random data within this subspace by drawing from a multi-dimensional normal distribution. To do this we must first define the mean and covariance matrices for each class. If, for simplicity, we assume linearity, then we may think of our random subspace as being the Principal Component space, and the data should be uncorrelated in this space, so we set the off diagonal elements of the covariance matrix to be zero and it only remains to determine the variances. We do this in such a way as to reflect a general property of biological expression data where the first principal component captures the most variance, and subsequent principal component capture successively smaller variances. We model this property very simply by setting the variance in the *i*^*th*^, principal component direction to be equal to *e*^-(*i*-1)^, such that the variance in the first principal direction is 1, and in the second *e*^-1^ etc. We assign the same covariance matrix to both the “control” and “perturbed” samples. We choose the mean of the “control” samples to be zero, and the mean of the “perturbed” samples to reflect the pre-defined differentially expressed genes by setting it equal to $\hat{m}$ scaled by Δ to control the magnitude of the difference between the “control” and “perturbed” expression data. An illustration of the synthetic data generated in a low-dimensional space with the parameters *p* = 3, *n*_*d*_ = 2, *D* = 2, *N* = 3, and Δ = 3.0 with gene 1 and 3 chosen to be differentially expressed, is shown in Figure 3. These parameters were chosen to give an impression of the structure of the data in higher dimensions, and to result in a clear difference between the two classes of samples.

### Estimating significant DEG applied to the synthetic data

The Characteristic Direction method is represented by a vector in expression space, each component of which corresponds to a gene. We interpret this vector by taking the square of each component to be a measure of the importance of the corresponding gene in the differential expression; the larger the squared component the more significant the gene. In order to determine the appropriate threshold above which to accept genes as differentially expressed we derive a null distribution for the ranks of the squared components as follows:

Given a null hypothesis that there are no differentially expressed genes we would like to compare the distribution of squared component values to those that would be expected under the null hypothesis. One way to generate the null distribution would be to use permutations of the data, which would require sample sizes to be large enough to permit a sizable number of permutations. An alternative which does not require such large sample size is to use the same multivariate normal model of the data distribution used in the classification calculation to generate the null distribution. Under the null hypothesis we assume that there is no difference between the two classes of samples and that they both derive from a multivariate normal distribution with covariance matrix: Σ, the same as used in the classification calculation, and mean *m*_0_. We use the following algorithm to generate the null distribution of ranked squared coefficients:

1. Generate two random sample means by drawing from the multivariate student t distribution with *N* - 1 degrees of freedom and find their difference.

2. Calculate the null characteristic direction *b*_*null*_ = Σ^-1^ Δ_*m*_

3. Calculate $b_{\mathrm{null}}^{2}$ and rank the components into descending order of magnitude

4. Repeat steps 1–3 100 times, and take the mean, to give

$$b_{\mathrm{null}}^{2}$$

To compare the real distribution the null we take the ratio: $b^{2}/b_{\mathrm{null}}^{2}$. The simplest and most conservative approach would be to accept into the set of differentially expressed genes all those genes for which the ratio: $\frac{b^{2}}{b_{\mathrm{null}}^{2}}>1$. A less conservative method to derive the threshold from the data is to consider the inflection in the curves which can be isolated with the cumulative distributions.

### Performing characteristic direction enrichment analysis

**Figure 4:**
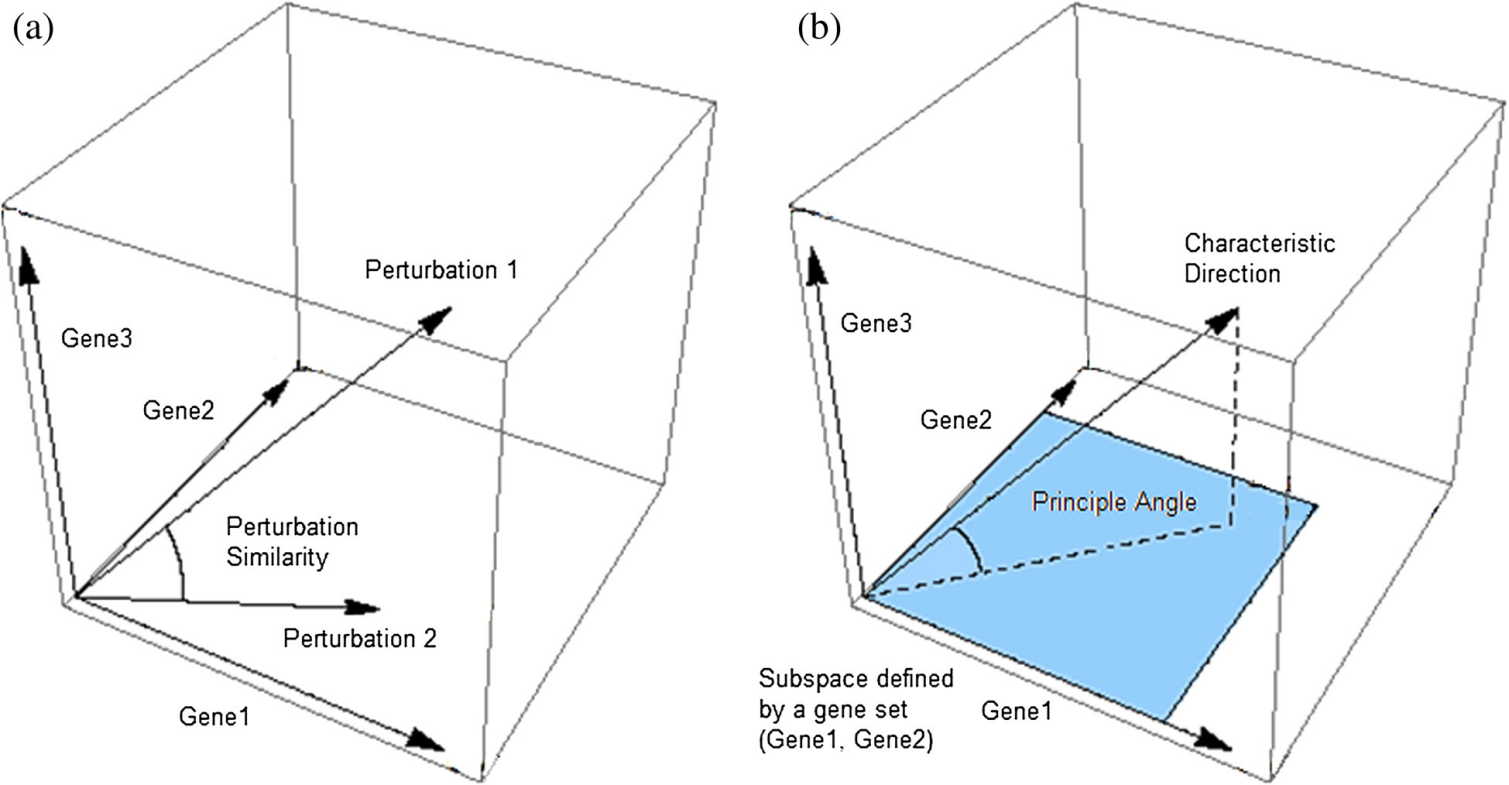
**Illustration of gene set enrichment with the characteristic direction concept. a)** Similarity between two perturbations can be interpreted as the angle subtended between two characteristic directions. **b)** Gene set enrichment analysis can be formulated as the principal angle between the characteristic direction and the subspace spanned by the genes in a gene set.

The geometrical picture of differential expression as a single direction obtained by the Characteristic Direction naturally leads to some extensions. The natural distance measure for two directions is the cosine distance, or equivalently, the angle between the two directions. In this way we can picture the similarity between two biological perturbations as the alignment between two directions (Figure 4a). Furthermore, a gene-set defines a subspace within expression space; we can use the angle subtended between this subspace and the direction characterizing the differential expression, the first principal angle (Figure 4b), as a quantitation of the significance of a pre-defined gene set for the differential expression. In the Additional file 1 we derive the appropriate null-distribution with which to compare this subtended angle, and with such a statistical test we can identify significantly differentially expressed gene-sets. In the Results section we compare this new method of enrichment analysis to existing methods such as gene set enrichment analysis (GSEA) [29,30] and find a suggestion that this new enrichment analysis leads to the inference of more relevant biological processes.

## Results

### Benchmarking the characteristic direction method with transcription factor perturbations followed by microarray genome-wide expression profiling

We collected 73 experiments from GEO (Additional file 1: Table S1) which contain expression data for control verses TF perturbation with at least three biological replicates in each of these classes. The TF perturbations consisted of knockdowns (32), knockouts (29), over-expressions (5), and other types of perturbation (7) such as partial mutations for example. A complete list with the details about these experiments can be found in the Additional file 1. We extracted processed expression values from the SOFT files downloaded from the GEO database. For each experiment, we compared control and perturbed classes with four different methods: the fold change, Welsh’s t test, SAM, and the geometrical approach described above which we shall refer to as the “characteristic direction” approach. Each experiment and method pair resulted in a ranked list of all genes on the particular array chip in order of their estimated significance in the differential expression.

To evaluate the ability of each method to prioritize DEG we used ChIP-Seq data reporting DNA binding sites for the each TF from one of two databases: ChEA [31] and ENCODE [32]. There is little overlap between these databases and so they constitute independent validations (see Additional file 1). Using this data we derived lists of genes which are associated with each TF by the identification that the TF bind to these genes’ promoters. Then, by assuming that genes from these lists are more likely to be regulated by the perturbed TF than the complementary genes, we reasoned that the degree to which an analysis method prioritizes the ChIP-Seq derived genes is a measure of its effectiveness. In addition, in a similar way we used lists of genes/proteins which are known to physically interact with each TF. We reasoned that genes for which their protein product physically interacts with the TF are more likely to be differentially expressed after the TF perturbation. As a final comparison, we examined the priority given to the perturbed TF itself, since it is known that many TF tend to auto-regulate their expression.

We took two approaches to examining and displaying the distributions of the rankings of these gene lists by the various methods: the cumulative distribution function over all experiments; and the distribution of the area under the curve (AUC) scores from each experiment. Before proceeding, we describe these two methods in more detail. Expression data from each experiment *E*_*j*_, with a total number of genes *p*_*j*_, is analyzed for differential expression, according to one of the methods described above, resulting in rankings for each gene which are scaled by *p*_*j*_ to give *r*_*ji*_, the scaled rank of gene *i* in experiment *j*, such that a value of *r*_*ji*_ = 0 is taken by the most significant gene in experiment *E*_*j*_ and *r*_*ji*_ = 1 is taken by the least significant gene. For each experiment we have a corresponding subset of genes *S*_*j*_ which may, for example, consist of genes which are putative target genes of the TF that was knocked down in the specific experiment, as determined by an independent ChIP-Seq experiment. We examine the rankings of the genes *S*_*j*_. The set of rank values of the genes *S*_*j*_ corresponding to experiment *E*_*j*_ are identified for all *j*,

(6) $$A=\cup_{j,k\in S_{j}}r_{\mathrm{jk}}$$

Then the cumulative distribution function of *A*, which we label *D*(*r*), is examined. If the gene sets *S*_*j*_ contain genes which are neither preferentially significant or insignificant then we expect a uniform distribution and

(7) $$D\left(r\right)=r$$

Any significant deviation from this indicates that the gene sets are significant in the differential expression analysis, therefore we examine *D*(*r*) - *r* for significant deviations from zero in order to evaluate the various methods. A significant positive value corresponds to the genes in *S*_*j*_ being concentrated at the smaller scaled ranks and therefore having greater significance than a uniform random distribution. The entire process is visualized in Figure 2.

Random fluctuations from zero are to be expected and we can estimate the scale for these fluctuations, *φ*, by a premise similar to that behind the Kolmogorov-Smirnov test (see the Additional file 1 for details). When plotting *D*(*r*) - *r* we also include a right-hand scale to the plots which have the values scaled by *φ* to give an impression of how the deviation compares to what might be expected from random fluctuations under the null hypothesis of a uniform distribution of rankings. Values >> 1 on this scale indicate significant non-uniformity in the distribution of ranks.

**Figure 5:**
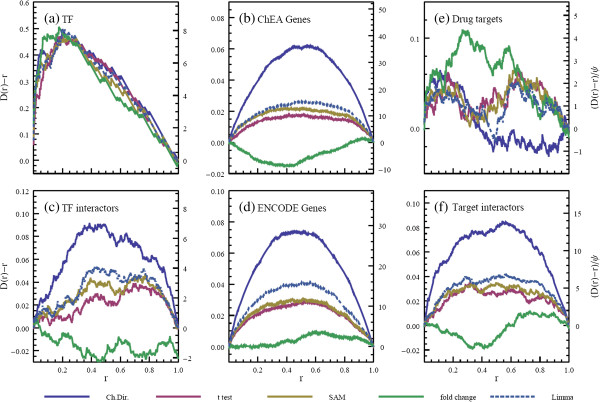
**Comparison of the distributions of the scaled rankings of the gene sets for the various methods for the TF (a-d) and drug (e-f) perturbations.** Each sub-plot shows the deviation of the cumulative distribution from uniform of the rankings of each gene set and analysis method, **(a)** the TF perturbed by each experiment; **b)** genes associated with binding sites of the TF as measured in ChIP-Seq experiments from ChEA; **c)** the genes interacting with the TF or the gene that encodes the TF; **d)** genes associated with binding sites of the TF as measured in ChIP-Seq experiments from ENCODE; The perturbation of the cumulative distribution of the rankings of **(e)** drug targets, and **(f)** genes that their protein product are known to interact with the drug targets.

Hence, this method allows us to visually and quantitatively compare the perturbation of cumulative distribution functions from uniform, *D*(*r*) - *r*, for each ranking method and each gene list type (Figure 5a-d). Apart from Figure 5a, which shows that all the methods are equally able to identify the TF directly perturbed in each experiment, the relative performance of the methods are quite consistent across the gene lists. The Characteristic Direction method prioritizes genes in the differential expression which are also associated with the perturbed TF in ChIP-Seq data, and also genes which interact with the TF, and it does so to a significantly higher degree than the other methods (Kolmogorov-Smirnov test p values comparing all the distributions can be found in Additional file 1: Tables S3 to S6). Limma is the next best performing method by this measure, followed by SAM and Welsh’s t test. The fold change method does not seem to successfully prioritize the gene list. We also found that the degree of shrinkage has little effect on the rankings generated by the Characteristic Direction approach and thus choose a representative value (*y* = 1).

### Benchmarking the characteristic direction method with drug perturbations followed by microarray genome-wide expression profiling

Next we collected 130 experiments from GEO (Additional file 1: Table S2) consisting of control verses FDA approved drug perturbed samples, with at least three biological replicates in each sample. The genes were ranked in the same way as in the previous subsection, using the same methods. Due to the different mechanisms of action between TFs and drugs, instead of using ChIP-Seq for validation we assessed the rankings of known drug targets, and separately, genes which are known to have their protein products directly physically interact with those drug targets using known protein-protein interactions available from the NCBI Gene database and drug targets from DrugBank [33]. We assess the prioritization of the genes with the DEG calling methods in the same way as for the TFs otherwise. It should be noted that ChIP-Seq data informs the validation with relatively unbiased and objective data whereas the knowledge of drug targets is rather more biased and incomplete. In addition, it is not known whether targeting a drug target with a drug will alter the mRNA expression of the target. So we do not expect to see the same strength of signal in this form of validation as compared with the validation for TF perturbation followed by expression with ChIP-Seq prior data. The performance of each method seems to be in the same relation as for the TFs, with the characteristic direction giving higher priority to the genes encoding drug targets of the relevant drugs and genes which their products interact with those targets (Figure 5e-f) (Kolmogorov-Smirnov test p-values comparing all the distributions can be found in Additional file 1: Tables S7 and S8).

### Comparing the characteristic direction method to DESeq

**Figure 6:**
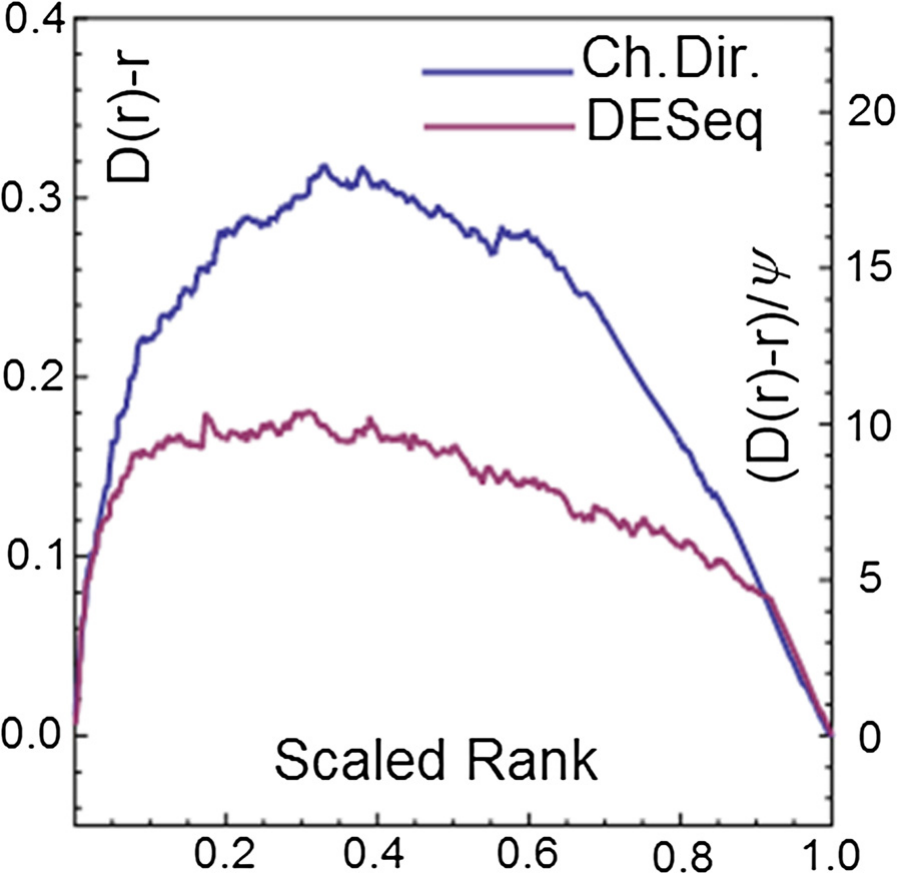
Distribution of the top 500 genes associated with differential STAT3 binding in the ranked list of differentially expressed genes as determined by DESeq or the characteristic direction.

In a recent study Hardee et al. [34] studied the relationship between differential STAT3 binding and differential gene expression in two subtypes: germinal center B-cell-like (GCB) and activated B-cell-like (ABC) of diffuse large B-cell lymphoma (DLBCL). The Illumina Genome Analyzer IIx high-throughput sequencing platform was used to perform ChIP-Seq experiments identifying the DNA binding of the TF STAT3 and also RNA-Seq experiments were performed on eight patient-derived cell lines: four from each subtype of DLBCL. The binding of STAT3 was studied because deregulation of this TF is known to be an important discriminant between the two subtypes of cancer. The ChIP-Seq data was condensed into 10337 binding regions (BR) for each cell line and the authors identify differential binding of STAT3 between the two subtypes using DESeq analysis. In addition, the authors identify differentially expressed genes, again using DESeq. One of the central findings of their work is that there is a strong relationship between the differential binding of STAT3 and the differential gene expression between the two subtypes of BLBCL. Stated another way, genes associated with binding regions which are differentially bound by STAT3 also tend to be identified as differentially expressed. This study provides an opportunity to compare the performance of the Characteristic Direction approach in the setting of deep sequencing technologies to one of the most popular differential expression methods in the field (DESeq). To do this, we repeated the differential analysis of both the ChIP-Seq data and the RNA-Seq data, but where the authors used DESeq, we use the Characteristic Direction; we then re-examined the association between differential STAT3 binding and differential gene expression. Taking the top 500 genes associated with differential STAT3 binding as determined by the Characteristic Direction and DESeq respectively, we examined the distribution of the DEG in the same way as we did for the TFs (Figure 6). We see that the Characteristic Direction results in a higher ranking of the genes associated with differential binding of STAT3. More genes associated with differential binding of STAT3 are recovered from the differential expression analysis when the Characteristic Direction method was used. The result further demonstrates the greater apparent degree of consistency between DNA binding data and differential expression analysis uncovered when using the Characteristic Direction.

### Benchmarking the characteristic direction method with synthetic data

**Figure 7:**
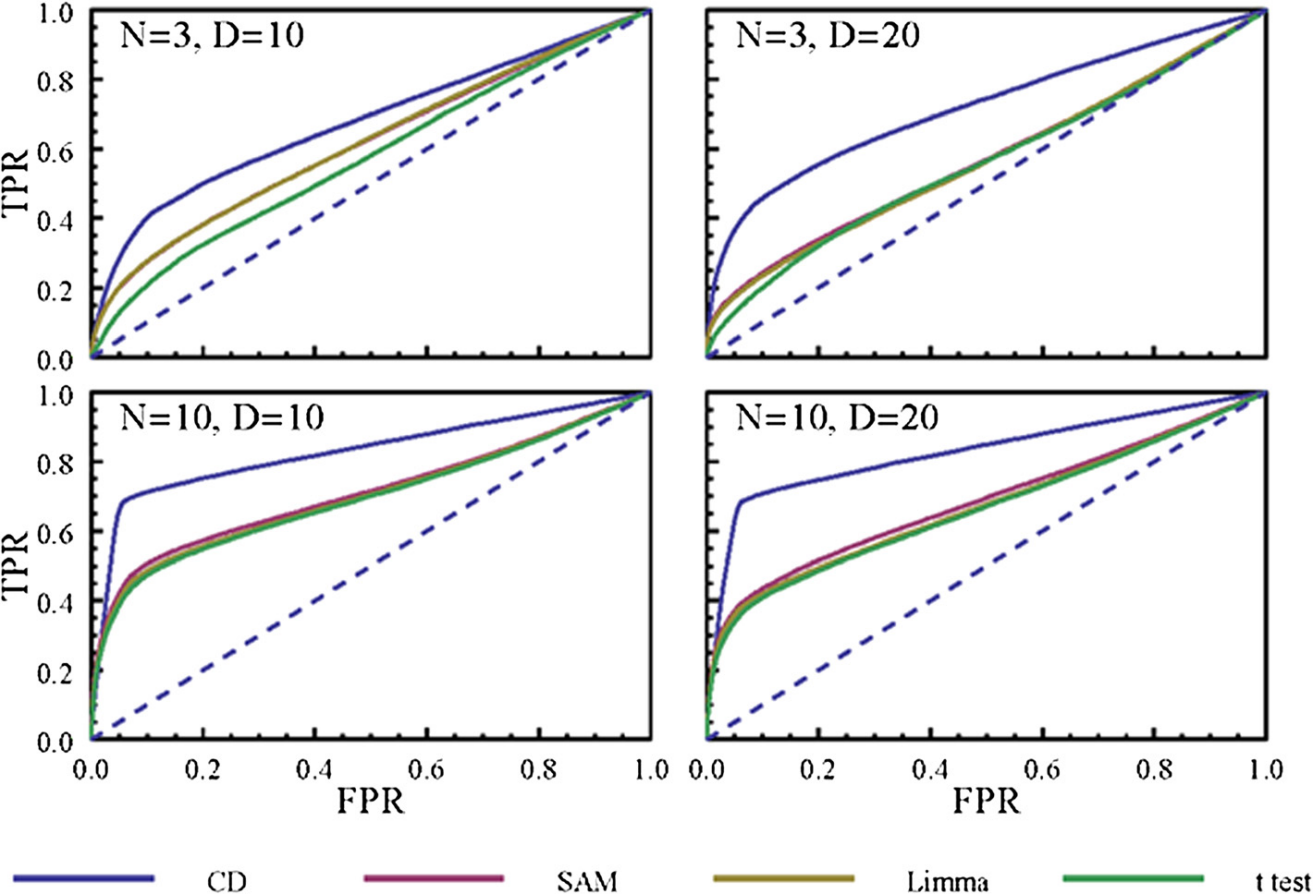
**ROC curves comparing the various DEG ranking methods for the ability to identify DEG from synthetic data created by the following parameters: *p *** **= 10**^**4**^**, *n***_***d***_**= 2 × 10**^**3**^**, and ∆=0.3; the remaining parameters are as indicated in the figure panels.**

We used the following parameters to generate synthetic data as described in the Methods: *p* = 10^4^, *n*_*d*_ = 2 × 10^3^, as these are of the same order of magnitude of whole genome profiles and we used Δ = 0.3 as this resulted in data for which it was not too difficult and not too easy to identify the differentially expressed genes. We repeated each simulation 10 times. We investigated two different values of the sample size (3 and 10) as these are two common sample sizes found in GEO datasets, and we also examined two different values for the dimensionality (10 and 20). The resulting ROC curves show that the characteristic direction outperforms the other methods in recovering the differentially expressed genes from the synthetic data (Figure 7).

### Estimating significant DEG applied to the synthetic data

**Figure 8:**
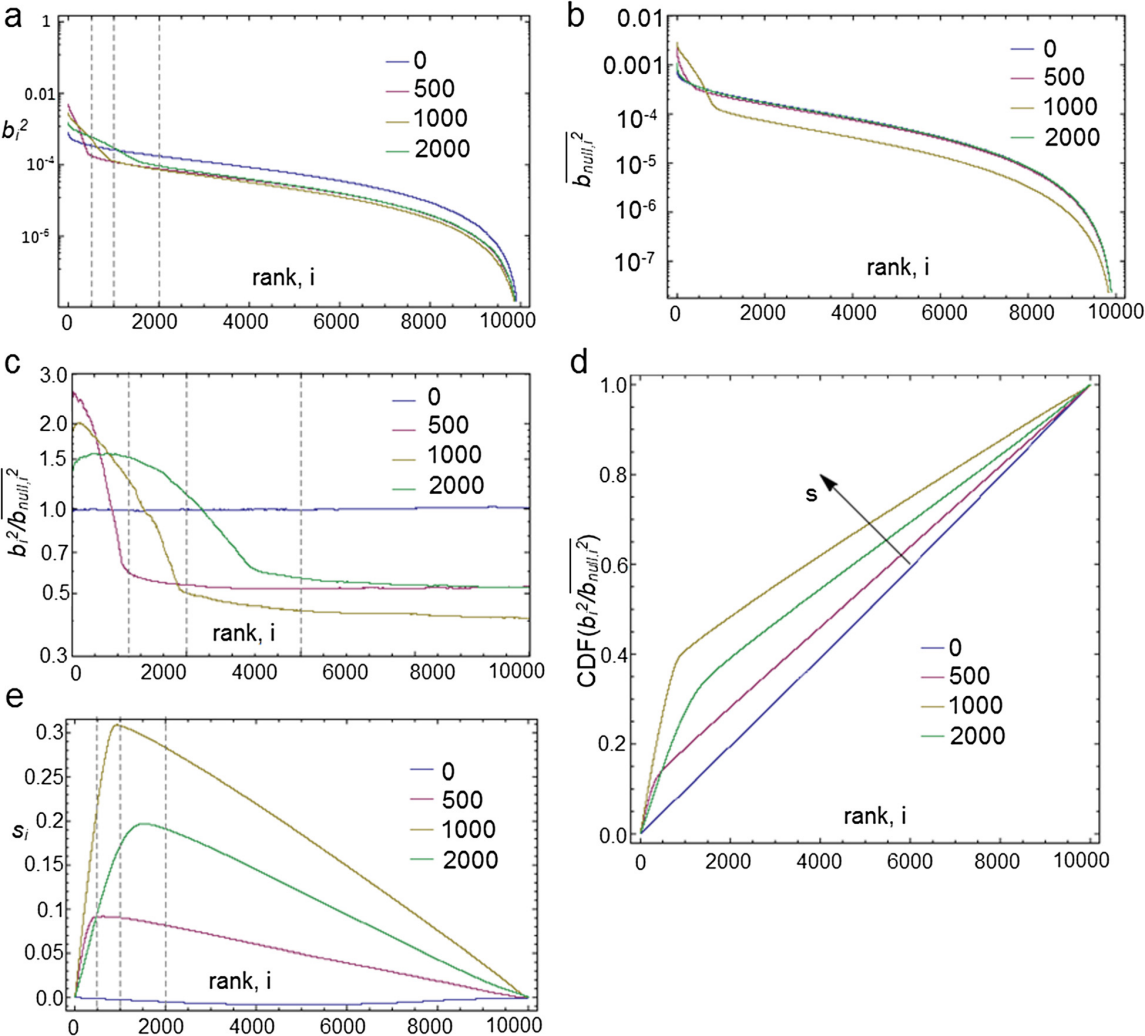
**Deciding where to place the cutoff using synthetic data. a)** Sorted squared characteristic direction components for the various synthetic datasets. Dashed lines indicate the top 500, 1000, and 2000 genes. **b)** The null ranked squared coefficient distribution for the synthetic datasets. **c)** The ratio of the ranked squared coefficient distribution for the synthetic datasets to the null distribution assuming no difference between the classes. Dashed lines indicate the top 500, 1000, and 2000 genes. **d)** The cumulative distribution of the ratio between the squared coefficient distribution and the null distribution. The variable, s, which is indicated with an arrow, measures the distance perpendicular to the diagonal. **e)** The value of s for each of the synthetic datasets. The dashed lines indicate the top 500, 1000, and 2000 genes.

In many cases prioritization of differentially expressed genes is not the only aim – a discrete list of genes which are to be regarded as differentially expressed is required. We next use the synthetic data described above in order to demonstrate an approach for setting a threshold that would determine significant differential expression. Unless stated otherwise, all results in this section are averaged over three datasets. We first create a synthetic dataset where the number of differentially expressed genes is either 0, 500, 1000 or 2000. The set of genes we aim to identify as differentially expressed genes are those with particularly large squared components. However, by plotting the ranked squared components for each gene it is not clear where it would be appropriate to apply a threshold (Figure 8a). By applying the method that creates a null distribution for these ranked components, based on the null hypothesis that there are no differentially expressed genes, as described in the Methods (Figure 8b), we notice that when there are no differentially expressed genes the scaled components are uniformly distributed, with no components standing out (Figure 8c). However, when there are differentially expressed genes we observed a peak with a width which reflects the number of differentially expressed genes in the synthetic data (Figure 8c). By examining these curves we should be able to see whether there are any differentially expressed genes at all and if so how many; we therefore take the approach of allowing the data to decide the threshold as described in the Methods using two types of thresholds: one stringent and the other less stringent.

**Figure 9:**
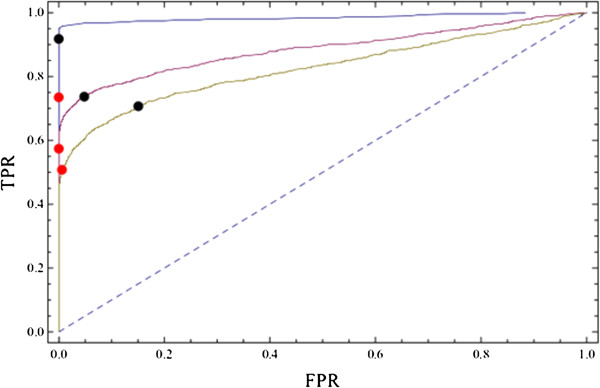
**ROC curves for the synthetic datasets with points indicating the FPR and TPR values at the various thresholds.** Red points show the values for the more conservative threshold value of b^2=b_null^2 and the black points indicate the values correspond to the peak of the curves in Figure 8.

The points on the curve which are closest to the top left corner capture more of the total differential expression with fewer genes. We label a new coordinate: *s*. which is perpendicular to the diagonal, and plot its value for each of the synthetic datasets (Figure 8d). The peaks of these curves correspond to the inflections in the curves in Figure 8c. Their height indicates the degree of differential expression – values which are a significant fraction of unity indicate a significant differential expression (Figure 8e). Note that this criterion is satisfied by all the synthetic datasets shown with the exception of the dataset with no differentially expressed genes. The position of the peak may also be taken as the threshold for acceptance into the set of differentially expressed genes. Finally, we indicate the position of the thresholds on ROC curves to demonstrate that we have indeed found good thresholds for identifying DEG (Figure 9). The sets of differentially expressed genes thus identified have sensible values of the false and true positive rates while also having the advantage that they are derived from the data itself rather than from the application of an arbitrary threshold.

### Characteristic direction enrichment analysis

In the case study presented in this section we attempt to compare the various biological contexts that emerge when examining differentially expressed genes identified from mRNA profiling of CD44+ CD24-/low breast cancer cells as compared with normal breast epithelium tissue. The data used in this case study for evaluation and validation comes primarily from a study that profiled and compared normal breast epithelium tissue obtained from reduction mammoplasties and highly tumorigenic breast cancer cells isolated from tumors (ESA+ CD44+ CD24-/low Lin-) [35]. The various approaches to identify DEGs from this dataset may provide different pictures of the biological mechanisms which are relevant to the disease. When comparing CD44+ CD24-/low breast cancer stem cells with normal breast epithelium tissue we expect to detect biological processes such as cell motility, cell proliferation, wound healing [36], and extra cellular matrix (ECM) remodeling which are known to be up-regulated in cancer stem cells and are activated in aggressive tumors.

**Figure 10:**
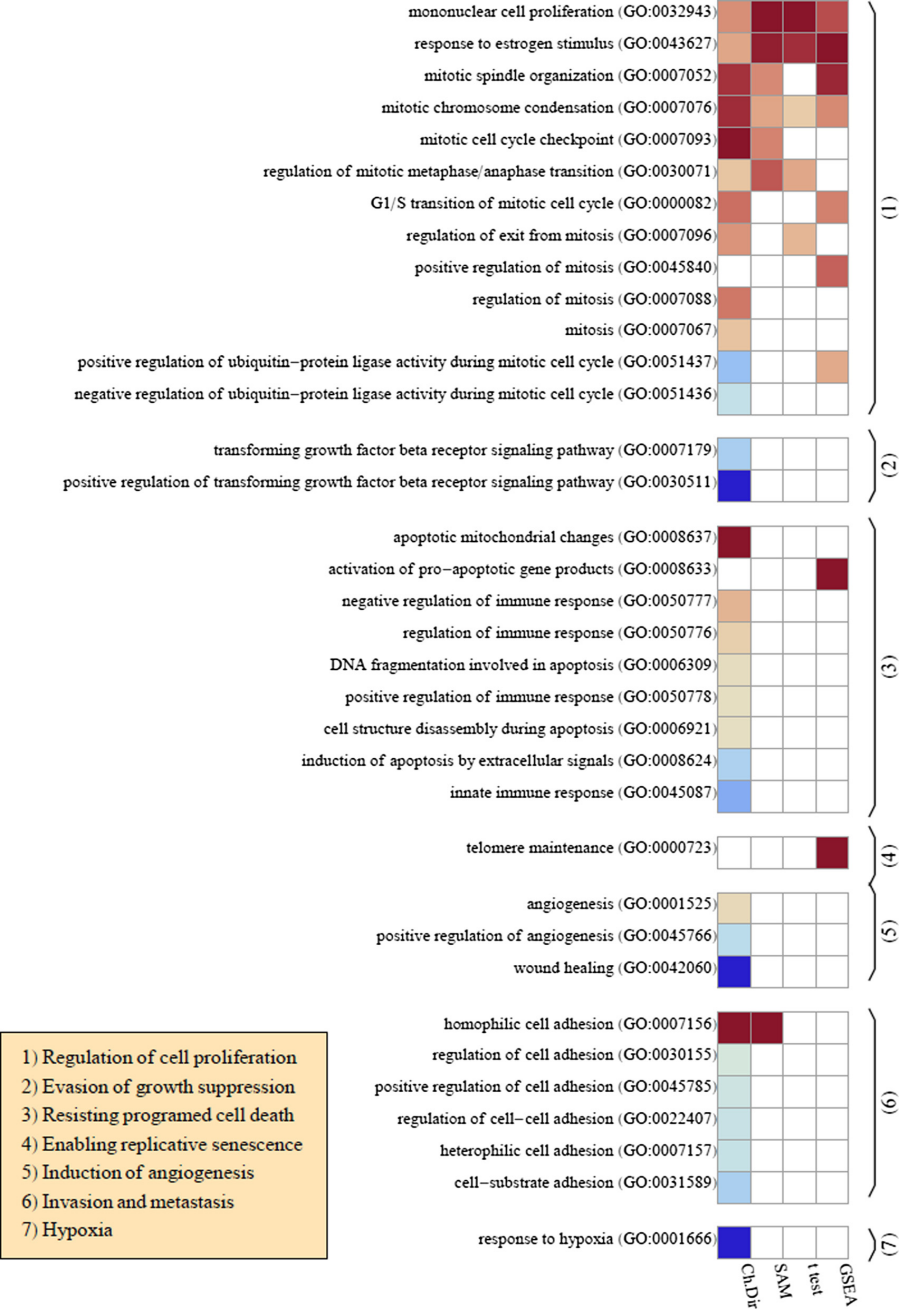
**Comparison of hallmark GO biological processes identified as significant in the differential expression of tumorigenic verses normal samples by enrichment of the significant genes called by various the methods.** Results of GSEA [15,30] analysis are included for comparison. Colored boxes indicate that the GO category is identified as significant with an FDR of 10% (60% for GSEA), and deeper red colors have a smaller mean rank of the gene set, corresponding to more up-regulation of the set, while deeper blue colors have a larger mean rank, corresponding to more down-regulation of the set. The GO categories are sub-categorized corresponding to the six hallmark characteristics of cancer as indicated in the inset box. The seventh category is included to evaluate the significance of the hypoxia GO category.

One commonly used approach to obtain a picture of the biology from the analysis of differential expression is the evaluation of the DEG for enrichment given previously annotated gene sets. Gene Set Enrichment Analysis (GSEA) mentioned in the introduction, is one of the most popular approaches to accomplish this task. A more basic and widely used approach is to use Welsh’s t test, or SAM, to identify differentially expressed genes and then apply the Hypergeomtric test to examine enrichment of gene sets deriving from various gene-set libraries or the Gene Ontology [37]. We can use these methods, as well as the characteristic direction approach to evaluate and compare significant biologically meaningful gene sets. We first manually construct six subsets of GO biological processes corresponding to the six hallmark characteristics of cancer as defined by Hanahan and Weinberg [38]: 1) regulation of cell proliferation; 2) evasion of growth suppression; 3) resisting cell death; 4) enabling replicative senescence; 5) induction of angiogenesis; and 6) enabling invasion and metastasis [38,39]. We then performed enrichment analyses for genes involved in these GO biological processes, using the DEG obtained with each of the methods and compared the resulting picture of the biology that develops in each case (Figure 10). In the case of the characteristic direction, enrichment was calculated using the geometrical concept of the principal angle described above (see Additional file 1 for more detail). In the case of Welsh’s t test and SAM a representative FDR threshold was set, resulting in a set of DEG and the significance of the overlap of the DEG with GO biological process gene sets was evaluated with the hypergeometric test with an FDR threshold of 10%. We also include the results of using GSEA [15], though we found it necessary to increase the FDR threshold to the rather larger size of 60% in this case in order to observe a comparable number of significant processes. We observe complete agreement between all the methods in the GO categories of mononuclear cell proliferation (GO: 0032943) and response to estrogen stimulus (GO: 0043627); cell proliferative processes are known to be fundamental to carcinogenesis and estrogen signaling is known to play a significant role in breast cancer. The characteristic direction approach with principal angle enrichment finds more processes to be significant in the differential expression in all the hallmark categories. It is possible that this is because this approach leads to a clearer picture of the differential expression, however, it would require further exploration before a more categorical statement can be made on this matter.

## Discussion and conclusions

We have described a new multivariate approach to differential expression which is better able to identify DEG while also addressing the issues associated with the high dimensionality of expression data. The Characteristic Direction approach uses the orientation of the separating hyperplane from a linear classification scheme, linear discriminant analysis, to define a direction which characterizes the differential expression. This results in a simple, highly-regularized characterization which is appropriate for genome-wide expression analysis. We compared the performance of this approach to established univariate approaches, with real and synthetic data. The validation scheme in the context of TF and drug perturbations is in itself valuable for benchmarking both computational and experimental methods. Extracting a large number of control verses perturbation expression datasets from GEO and prioritizing the genes with the various methods, we were able to show that the Characteristic Direction approach prioritizes genes which are associated with the binding sites of the perturbed TF and targets of drugs respectively; and it does so to significantly greater degree than a selection of popular methods. We took advantage of the opportunity to use independent prior knowledge datasets to validate our method. It is established that binding and unbinding of transcription factors to the promoters of genes is, in general, used for gene expression regulation. However, it is also clear that binding to the promoter does not necessarily result in differential expression. This is especially true when considering different cellular contexts. In most cases of our validation scheme the ChIP and array do not come from the same cell lines. However, there is some correlation/overlap between DEG after TF knockdowns and TF putative binding based on ChIP for the same TF in most cases. We do not know the true positives but we know that more overlap is likely due to a more accurate method to identify the DEG. We name this a silver standard for validation as it is not as good as a gold standard but it is good enough to compare DEG calling methods. The fact that we were able to recover genes associated with the binding sites of the perturbed TF is interesting on its own as it reveals a relation between DNA interactions identified by ChIP-Seq experiments and mRNA levels from expression profiling. Similarly, the ability of the method to identify a clear relationship between drug targets and the differential expression of their interactors in a systematic way is also noteworthy because for many drugs we do not know the targeted pathways while differential expression signatures are readily available. For the RNA-Seq validation we used a single study which compares differential binding of a TF, to differential expression, in the context of high-throughput sequencing. We found a stronger apparent relationship between differential binding and differential expression when using the Characteristic Direction approach as compared to the DESeq method. Like all statistical methods, the Characteristic Direction method works best when there are many repeats of the same condition. In principle, the method requires at least two repeats, but at least three repeats are needed for practical applications. The microarray and RNA-seq data used for validation of the method always had at least three repeats for each condition. It is true that in most RNA-seq studies so far investigators do not have that many repeats (1 or 2), but this is likely to change as the cost of such experiments rapidly drops. To make the Characteristic Direction method accessible, we implemented it in Python, R, MATLAB and Mathematica. Readers that are interested in applying the method to their own data should refer to the open source scripts and examples available at: http://www.maayanlab.net/CD.

### Availability and requirements

Implementations of the method are provided in Python, R, MATLAB, and Mathematica freely available at: http://www.maayanlab.net/CD.

## Competing interests

The authors declare that they have no competing interests.

## Authors’ contributions

AM and NRB designed the study and wrote the paper. NRB performed all analyses, wrote all the equations and implemented the method in Mathematica. KH developed the TF perturbation gene-set library from GEO, ASF developed the drug perturbation gene-set library from GEO, YK processed the data from ENCODE, EYC implemented the method in Python, QD implemented the method in MATLAB and R. All authors read and approved the final manuscript.

## Supplementary Material

Additional file 1 The Characteristic Direction: A Geometrical Approach to Identify Differentially Expressed Genes. Click here for file
