# Supplementary material for: The characteristic direction: a geometrical approach to identify differentially expressed genes
Source: BMC Bioinformatics. 2014 Mar 21;15:79. doi: 10.1186/1471-2105-15-79 (PMC4000056; doi:10.1186/1471-2105-15-79)
Supplement: Additional file 1 — The Characteristic Direction: A Geometrical Approach to Identify Differentially Expressed Genes. [file 1471-2105-15-79-S1.pdf]

Supplementary Materials for:

The Characteristic Direction: A Geometrical Approach to Identify Differentially Expressed Genes

Neil R. Clark[1]
Email: neil.clark@mssm.edu

Kevin Hu[1]
Email: kevin.hu@mssm.edu

Axel S. Feldmann[1]
Email: axel.s.feldmann@gmail.com

Yan Kou[1]
Email: yan.kou@mssm.edu

Edward Y. Chen[1]
Email: edychen@gmail.com

Qiaonan Duan[1]
Email: qiaonan.duan@mssm.edu

Avi Ma'ayan[1,*]
[*]Corresponding author
Email: avi.maayan@mssm.edu

[1]Department of Pharmacology and Systems Therapeutics, Systems Biology Center New York (SBCNY), Icahn School of Medicine at Mount Sinai School, New York, NY 10029 USA

Supplementary Text

The supplementary method text is split into two main parts: the first part deals with the mathematical details of the characteristic direction approach, and the second deals with the details of the validation.

Detailed Mathematical derivation of the characteristic direction

Suppose we have gene expression data from a number of samples N, in which the expression of p genes is measured, and then let each microarray profile form a row of the matrix $\boldsymbol{X}$ (an $N \times p$ matrix). For generality at this point we shall consider the case where each of the microarray samples comes from one of K classes belonging to the set G.
Let $f_k(x)$ be the class-conditional density of X, where x refers to a particular instance of the values in a gene expression profile, and let π_k be the prior probability of class k. Bays rule provides an expression for the class posteriors $P(G|X)$,

$$\Pr(G = k|X = x) = \frac{f_k(x)\pi_k}{\sum_{l=1}^{K} f_l(x)\pi_l}$$

In many circumstances it is reasonable to model the class-conditional density as a multivariate Gaussian, with mean μ_k, and covariance Σ_k,

$$f_k(x) = \frac{1}{(2\pi)^{\frac{p}{2}}|\Sigma_k|^{\frac{1}{2}}} e^{-\frac{1}{2}(x-\mu_k)^T \Sigma_k{}^{-1}(x-\mu_k)}$$

In linear discriminant analysis the assumption that the covariance matrix is the same for each class, $\Sigma_k = \Sigma \ \ \forall k$, is made. The log-ratios of two class posteriors then provides a measure of the relative likelihood of classifying to those classes; so the log ratio of classifying to classes k and l is given by,

$$log\frac{\Pr(G = k|X = x)}{\Pr(G = l|X = x)} = log\frac{f_k(x)}{f_l(x)} + log\frac{\pi_k}{\pi_l}$$
$$= log\frac{\pi_k}{\pi_l} - \frac{1}{2}(\mu_k - \mu_l)^T \Sigma^{-1}(\mu_k - \mu_l)$$
$$+ x^T \Sigma^{-1}(\mu_k - \mu_l)$$

This equation is linear in x, so the decision boundary between two classes is a hyper-plane, the orientation of which is defined by the normal vector,

$$b = \Sigma^{-1}(\mu_k - \mu_l)$$

We interpret this orientation to infer the properties of the differential expression.
The terms in the above expression for b are estimated from the data as follows:

- $\hat{\mu}_k = \sum_{g_i=k} \frac{x_i}{N_k}$
- $\hat{\Sigma} = \sum_{k=1}^{K} \sum_{g_i=k} (x_i - \hat{\mu}_k)\,(x_i - \hat{\mu}_k)^T / (N - K)$

Where x_i is a row from the data matrix $\boldsymbol{X}$, and g_i is the class of the expression profile in row i.

The dimensionality problem

When $p \gg N$, as is the case for genome-wide expression profiling, the $p \times p$ covariance matrix has a rank at most equal to N, and therefore is singular. The calculation of the orientation vector b involves the inversion of this singular matrix. Regularization can be used to deal with this issue [1, 2]. Linear discriminant analysis can be regularized by shrinking the covariance matrix to the scalar variance σ^2 ,

$$\hat{\Sigma}(\gamma) = \gamma\hat{\Sigma} + (1-\gamma)\sigma^2 I_p, \qquad \text{with } \gamma \epsilon [0,1]$$

where I_p is the $p \times p$ identity matrix. Note a value of $\gamma = 1$ gives the original covariance estimate, a value of $\gamma = 0$ results in a diagonal covariance estimate with each variance value being shrunk towards the scalar variance σ^2 , and intermediate values of γ result in a mixture of the two. The inclusion of a constant on the diagonal is one way of resolving the singularity of the covariance matrix, and this type of regularization has been show to lead to more stable predictions.

Computational short-cut when $p \gg N$

To calculate the orientation vector b we must invert the (shrunken) covariance matrix which has dimensions $p \times p$. For genome-wide expression profiling the number of features may be extremely large and so the dimensions of the covariance matrix can be huge. However, when $p \gg N$ there is a computational shortcut which can be used [3] Because N points in a p-dimensional space span a subspace which has dimension at most $N-1$, it can be shown [1] that models (which do not use non-quadratic regularizing penalty terms) which are fit in this relatively low-dimensional subspace are equivalent to the same model fit in the full space. Models to which this theorem applies include linear discriminant analysis. Computing the model in the subspaced spanned by the data confers a large computational saving when $p \gg N$. Stated more explicitly: we can represent the data matrix in its singular-value decomposition,

$$\begin{aligned} X &= UDV^T \\ &= RV^T \end{aligned}$$

Where U is an $N \times N$ orthogonal matrix, D is a rectangular $N \times p$ diagonal matrix with diagonal elements $d_{11} \geq d_{22} \geq d_{NN} \geq 0$, which are termed the singular values, and V is a $p \times p$ orthogonal matrix. R is an $N \times N$ matrix of which the first $Rank(X)$ column are non-zero vectors. The theorem states that when fitting a model of this data the p-vectors x_i can be replaced by the N-vectors r_i, which are the rows of R, and the resulting coefficients will be the same after transformation by V. The normal vector to the separating hyper-plane is given by equation (equation for b), we first use the shrunken covariance matrix to rewrite it as,

$$b = \hat{\Sigma}^{-1}(\gamma).(\mu_k - \mu_l)$$

We use, $\Sigma = \frac{1}{N}X^T X$ in (equation for shrunken covariance matrix), to express the invers of the covariance matrix as:

$$\hat{\Sigma}^{-1}(\gamma) = \left(\gamma \frac{1}{N} X^T X + (1-\gamma)\sigma^2 I_p\right)^{-1}$$

Then we use the singular value decomposition in equation to express this as:

$$\hat{\Sigma}^{-1}(\gamma) = \left(\gamma \frac{1}{N} V^T R^T R V + (1-\gamma)\sigma^2 I_p\right)^{-1}$$

and with a little manipulation this becomes:

$$\hat{\Sigma}^{-1}(\gamma) = V\left(\gamma \frac{1}{N} R^T R + (1-\gamma)\sigma^2 I_p\right)^{-1} V^T$$

In order to gain further computational savings we notice that the above decomposition is equivalent to the principal component decomposition, where the columns of V are the principal component directions (sometimes referred to as loadings) and R is the matrix of scores. We take advantage of an efficient iterative algorithm for computing the principal component directions and scores which is called the Non-linear iterative partial least squares (NIPALS) algorithm. A brief description of the NIPALS algorithm is given in the following subsection.

We note that a truncated version of the scores and loading matrices, R_t and V_t can be used to decompose the data matrix to arbitrary accuracy. To avoid numerical issues arising from small singular values we truncate the principal components to a number, n_{PCA}, such that,

$$\frac{\sum_{i=1}^{n_{PCA}} d_{ii}}{\sum_{i=1}^{N} d_{ii}} \geq 1 - \varepsilon$$

where $\varepsilon \ll 1$ such that the great majority of the variance in the data matrix is preserved in the decomposition. After truncation the dimensions of the matrices change as follows,

$$R \to R_t: (N \times p) \to (N \times n_{PCA})$$
$$V \to V_t: (p \times p) \to (p \times n_{PCA})$$

where $n_{PCA} < N$. Then we us the truncated scores and loadings in equation (equation for invers sigma above) to finally write:

$$\hat{\Sigma}^{-1}(\gamma) = V_t\left(\gamma \frac{1}{N} R_t^T R_t + (1-\gamma)\sigma^2 I_{n_{PCA}}\right)^{-1} V_t^T$$

And we see that we do not need to invert the very large $p \times p$ matrix directly but we only need invert an $n_{PCA} \times n_{PCA}$ matrix. Our final calculating for the orientation of the hyper-plane after regularization is then:

$$b(\gamma) = V_t\left(\gamma \frac{1}{N} R_t^T R_t + (1-\gamma)\sigma^2 I_{n_{PCA}}\right)^{-1} V_t^T(\mu_k - \mu_l)$$

This can be calculated very efficiently using the NIPLAS algorithm to calculate R_t and V_t, with the number of operations reduced by this singular value decomposition trick from $O(p^3)$ to $O(pN^2)$ which is a significant saving when p is large and $p \gg N$, as is the case in genome-wide profiling.

More information on the singular value decomposition trick can be found in [1] and [2]. More information on Regularized linear discriminant analysis can be found in [1, 2] [4]

The Non-linear Iterative Partial Least Squares (NIPALS) algorithm [5]

The algorithm begins by mean-centering the data matrix X and variables are initialized by setting the matrix $E_{(0)} = X$, and the vector t equal to a random column of X. At the end of each iterative cycle (described in pseudo-code below) the vectors t and q will become individual scores and loadings. A parameter, h, which is used in the test of convergence of the iterative procedure, is set to a small value (typically $\sim 10^{-5}$). Finally the following steps are iterated through, and the process repeated for $i = 1$ up to the number of principal components to be calculated. The algorithm then runs through the following steps:

1) Project the data matrix X onto the vector, t, to obtain :
$$q = \frac{E_{(i-1)}^T t}{t^T t}$$
2) Normalize the loading vector:
$$q = \frac{1}{|q|} q$$
3) Project X onto q to and set:
$$t = \frac{E_{(i-1)} q}{q^T q}$$
4) Test convergence. If the difference between the eigenvalues $\tau_{new} = t^T t$ and τ_{old} from the previous iteration is larger than $h\tau_{new}$ then return to step 1
5) Remove the estimated principal component from $E_{(i-1)}$:
$$E_{(i)} = E_{(i-1)} - (tq^T)$$

Interpretation of the orientation of the separating hyper-plane

The p-vector b which is the normal to the separating hyper-plane is here interpreted as characterizing the differential expression. The normalized vector $\hat{b}$ contains only information about the direction of the normal to the separating hyper-plane. The components of $\hat{b}$ are the direction cosines, and their magnitude quantifies the degree of alignment of the direction normal to the separating hyper-plane to axes corresponding to each gene evaluated in the expression profile. The sign of each component can be interpreted as the sign of the contribution of each gene to the differential expression. Another way to picture this interpretation of gene significance is to consider the identity,
$$\sum_{i=1}^{p} \hat{b}_i^2 \equiv 1$$

Then the contribution of each $\hat{b}_i^2$ to this sum can be interpreted as quantifying the relative significance of the corresponding gene.

Calculation of the principal angle

We use a form of the algorithm described in [6] to calculate the single principal angle between a line, which has a direction parallel to the characteristic direction $\hat{b}$, and a subspace C defined by a gene set as the space spanned by each gene in the set.
The QR decomposition can be used to calculate orthonormal bases for each subspace,

$$Q_b = orth(\hat{b})$$

$$Q_C = orth(C)$$

where $Q_b \epsilon \mathbb{R}^{p\times 1}$, and $Q_c \epsilon \mathbb{R}^{p\times n_c}$ where n_c is the number of genes in the gene set. We then calculate the following singular-value decomposition,

$$[Y, W, Z] = svd({Q_b}^T Q_c)$$

Because one of our subspaces has dimension of unity there is only one non-zero value in W, which we call ω, and this is related to the single principal angle by:

$$\theta = \arccos(\omega)$$

This angle, which measures the alignment of the direction $\hat{b}$ with the subspace C defined by pre-defined gene set, is taken as a quantification of the enrichment of the gene set.

Calculation of the null-distribution of principal angles

We calculate an analytical expression which provides the null-distribution of the first principal angle between a line and an n-dimensional subspace. This is used to generate a p-value for the enrichment of gene sets. The null hypothesis is that the gene set is not enriched, and the angle between the corresponding subspace and the direction characterizing the differential expression is corresponds to an isotropic distribution. Therefore, we begin by calculating the distribution of the angles between a pair of isotropic directions. The surface area of an n-sphere is given by:

$$S_n(r) = \frac{2\pi^{\frac{n+1}{2}}}{\Gamma\left(\frac{n+1}{2}\right)} \qquad (11)$$

The probability distribution of the angle between two isotropic directions in an n-dimensional space is given by the ratio:

$$pdf(\theta) = \frac{S_{n-2}(Sin(\theta))}{S_{n-1}(1)} = \pi^{-\frac{1}{2}} \frac{\Gamma\left(\frac{n}{2}\right)}{\Gamma\left(\frac{n-1}{2}\right)} Sin^{n-2}(\theta) \qquad (12)$$

This can be generalized to find the probability distribution for the principal angle between a line and an m-dimensional subspace,

$$pdf(\theta) = \frac{S_{n-m-1}(Sin(\theta))S_{m-1}(Cos(\theta))}{S_{n-1}(1)} = 2\frac{\Gamma\left(\frac{n}{2}\right)}{\Gamma\left(\frac{n-m}{2}\right)\Gamma\left(\frac{m}{2}\right)} Sin^{n-m-1}(\theta)Cos^{m-1}(\theta) \qquad (12)$$

This can be integrated to give the cumulative distribution function:

$$cdf(\theta) = 2\frac{\Gamma\left(\frac{n}{2}\right)}{\Gamma\left(\frac{n-m}{2}\right)\Gamma\left(\frac{m}{2}\right)} \frac{1}{m} Cos^{m}(\theta)\ {}_2F_1\left(\frac{m}{2}, \frac{2+m-n}{2}, \frac{2+m}{2}, Cos^2(\theta)\right)$$

Where ${}_pF_q$ is the generalized hypergeometric function. This can be used to calculate the p value.

Validation methods

Collecting relevant datasets from GEO

Expression data was extracted from GEO GDS SOFT files (http://www.ncbi.nlm.nih.gov/gds/). The dataset value type is indicated in the file by the variable “!dataset_value_type”. The two types we encounter are “count” and “transformed count”. There is a small number of cases from both types which have negative expression values. We interpret these as having already being Log transformed and leave them unchanged. The “count” data types are log transformed, and the “transformed count” data types are left as they are, in this way we ensure that all the data we use are log transformed. We applied an exhaustive search for drug and transcription factor perturbation experiments without any consideration of the outcome.

Independence of the ChIP and ENCODE validation data sets

We compared the GMT lines from ChEA and ENCODE gene-set libraries we previously developed for the software Enrichr [3] by calculating the Jaccard distance between pairs of lines. A small number of lines were found to be identical, and these were removed from ChEA such that the two datasets constitute independent validations.

Univariate differential expression methods for comparison

Here we provide details of the methods used for finding DEG to which we compare our approach.

Welch t test

The Welsh t-test is a commonly used univariate approach to identify differentially expressed genes. The test is applied to each gene, i, independently, with sample means $\mu_{k,i}$ (which is equal to the ith element of the class mean μ_k), and sample variance $\sigma_{k,i}^2$ (which is equal to the ith diagonal element of Σ_k), and class sample sizes n_k, the test statistic for the difference between classes k and l is given by:

$$t_i = \frac{\mu_{k,i} - \mu_{l,i}}{\sqrt{\frac{\sigma_{k,i}^2}{n_k} - \frac{\sigma_{l,i}^2}{n_l}}} \qquad (9)$$

With the degrees of freedom given by,

$$df_i = \frac{\left(\frac{\sigma_{k,i}^2}{n_k} - \frac{\sigma_{l,i}^2}{n_l}\right)^2}{\frac{\sigma_{k,i}^4}{n_k^2(n_k-1)} + \frac{\sigma_{k,i}^4}{n_l^2(n_l-1)}} \qquad (10)$$

The p-value is then derived from the cumulative distribution function of the Student t distribution and p-values are commonly corrected for multiple hypotheses testing with the Benjamini-Hochberg test, resulting in equivalent q-values. We rank the genes by their significance.

Fold change

It is widely known that the fold-change is an insufficient statistic for the evaluation of differential expression [7, 8], however it is still commonly used and we include it here for comparison. This is a univariate characterization of differential expression, and for each gene, i, the log ratio of the class mean expression is calculated,

$$f_i = Log(\mu_{l,i}) - Log(\mu_{k,i})$$

The genes are then ranked by the magnitude, $|f_i|$, such that those genes which show the largest fractional change of mean value are regarded as the most significant according to this approach.

Significance Analysis of Microarrays (SAM)

Tusher et. al. in [9] describe the significance analysis of microarrays (SAM), which is a method for identifying genes on a microarray which show statistically significant changes in their expression (differentially expressed genes), essentially by assimilating a set of gene-specific t tests. This is another univariate approach to which we compare our method. We used the author provided R package to perform SAM analysis.

Limma

The authors of [10] detail the Limma method of differential expression analysis, which applies a linear model to the expression of each gene. We use the author provided R package to perform the Limma analysis.

Analysis of the ranking data

Each experiment consists of microarray profiles (after processing) from two classes, one is a control set and one is a set of samples after perturbation. We use these processed expression values in analysis of the differential expression with four different approaches (characteristic direction, Wech's t test, SAM, and fold change) to rank the genes by their significance. We then take lists of genes which may be expected to be significant for the perturbation and examine their positions in the ranked lists of all genes in each expression profile.

We took two approaches to examining and displaying the distributions of these rankings; the cumulative distribution function over all experiments, and the distribution of area under the curve (AUC) from each experiment. Here we describe these analyses in more detail.

Expression data from each experiment E_j, with a total number of genes p_j, is analyzed for differential expression (according to one of the methods described above) resulting in rankings for each gene which are scaled by p_j to give r_{ji}, such that a value of $r_{ji} = 0$ is taken by the most significant gene in experiment E_j and $r_{ji} = 1$ is taken by the least significant gene. For each experiment we have a corresponding subset of genes S_j (which may consist of genes associated with binding sites of the transcription factor knocked down in the experiment for example) whose rankings we wish to examine. The set of ranking of the genes S_j corresponding to experiment E_j are identified for all j,

$$A = \bigcup_{j,k \in S_j} r_{jk}$$

Then the cumulative distribution function of A, which we label $D(r)$, is examined. If the gene sets S_j contain genes which are neither preferentially significant or insignificant then we expect a uniform distribution and

$$D(r) = r$$

Any significant deviation from this indicates that the gene sets are significant in the differential expression analysis, therefore we examine $D(r) - r$ for significant deviations from zero in order to evaluate the various methods. A significant positive value corresponds to the genes in S_j being concentrated at the smaller scaled ranks and therefore greater significance than a uniform random distribution.

Random fluctuations from zero are to be expected and we can estimate the scale for these fluctuations, φ, by a premise similar to that behind the Kolmogorov-Smirnov test. If we hypothesis that, n, data points are drawn from a uniform distribution over $r \in [0,1]$, then we expect the resulting difference between the cumulative distribution function and r to be like a random walk fixed at zero at the points $r = 0$ and $r = 1$. We estimate the appropriate scale for the fluctuations by calculating the standard deviation of the deviation from 0 at the midpoint of the walk. At the midpoint of the walk, when $n/2$ steps have been taken, we have taken n_+ up-

steps and n_- down steps, and we have a displacement of x, then the following pair of equations hold:

$$n_+ + n_- = \frac{n}{2}$$
$$n_+ - n_- = x$$

Taking the sum of these two equations,

$$2n_+ = \frac{n}{2} + x$$

and we see that n_+ and x are linearly related. If we calculate the standard deviation for n_+ then we can use this linear relation to calculate the standard deviation of x. n_+ is distributed as a Hypergeometric distribution with a draw size of $n/2$, a number of successes equal to $n/2$, and a total of n, which has a variance of,

$$\frac{n}{16}\frac{n}{n-1}$$

In cases where $n \gg 1$ we shall approximate this with $n/16$. The standard deviation of n_+ is then given by:

$$\sqrt{\frac{n}{16}}$$

Then because of the above linear relation between $n_+$and x we need only double this to obtain the variance of the mid-point displacement from zero:

$$\sqrt{\frac{n}{4}}$$

Finally, given that the step size in the cumulative distribution is $1/n$, the scale for fluctuations is,

$$\varphi = \sqrt{\frac{1}{4n}}$$

When plotting $D(r) - r$ we also include a right-hand scale to the plots which have the values scaled by φ to give an impression of how the deviation compares to what might be expected from random fluctuations under the null hypothesis of a uniform distribution of rankings.

Supplementary Tables

	GEO ID	TF	Perturbation Type	TF present on array	Interacting genes present on arrray	TF present in ChEA	TF present in Encode
1	GDS1245	GATA1	KD	1	1	1	1
2	GDS1733	HSF1	KD	1	1	1	1
3	GDS1824	NANOG	KD	1	0	1	1
4	GDS1824	POU5F1	KD	1	1	1	1
5	GDS1942	KLF4	OE	1	1	1	0
6	GDS2069	KLF7	KO	1	1	0	0
7	GDS2088	TP63	KD	1	1	1	0
8	GDS2193	SOX4	KD	1	1	0	0
9	GDS2359	IRF6	KD	1	0	0	0
10	GDS2411	SPI1	KD	0	1	1	0
11	GDS2526	MYC	KD	1	1	1	0
12	GDS2629	GRHL3	KO	1	1	0	0
13	GDS2703	KLF9	KO	1	1	0	0
14	GDS2724	BMI1	KD	1	1	1	0
15	GDS2724	PCGF2	KD	1	1	0	0
16	GDS2747	WT1	KO	1	1	1	0
17	GDS2761	HIF1A	KD	1	1	1	0
18	GDS2761	HIF2A	KD	0	0	0	0
19	GDS2789	LMO4	DN	1	1	0	0
20	GDS2817	GLIS2	KO/VR	1	0	0	0
21	GDS3000	NEUROD1	KO	1	1	0	0
22	GDS3010	PLAGL2	KO	1	0	0	0
23	GDS3058	SP3	KO	1	1	0	0
24	GDS3106	STAT3	KD	1	1	1	1
25	GDS3171	TFAP2C	VR	1	1	1	0
26	GDS3173	EMX2	KO	1	0	0	0
27	GDS3178	BCL11B	KO	1	1	1	0
28	GDS3320	LMX1B	KO	1	0	0	0
29	GDS3385	STAT5B	KO	1	1	0	0
30	GDS3406	NFE2L2	KO	1	1	1	0
31	GDS3446	STAT3	OE	1	1	1	1
32	GDS3486	GATA4	KO	1	1	1	0
33	GDS3487	CREB1	KD	1	1	1	1
34	GDS3577	CBFB	KD	1	1	0	0
35	GDS3578	CTNNB1	KD	1	1	1	0
36	GDS3607	EGR1	KO	1	1	1	1
37	GDS3622	NFE2L2	KO	1	1	1	0
38	GDS3659	ZFPM2	KO	1	1	0	0
39	GDS3663	GATA4	KO	1	1	1	0
40	GDS3730	TARDBP	KD	1	1	0	0

41	GDS3732	SRF	KO	1	1	1	1
42	GDS3788	YY1	KD	1	1	1	1
43	GDS3788	YY2	KD	1	0	0	0
44	GDS3812	GLIS3	KO	1	1	0	0
45	GDS3912	CTNNB1	VR	1	1	1	0
46	GDS3926	HNF4A	KD	1	1	1	1
47	GDS4042	POU4F1	KO	1	1	0	0
48	GDS4058	SIRT3	KO	1	1	0	0
49	GDS4061	ESR1	KD	1	1	1	0
50	GDS4080	GATA3	OE	1	1	1	1
51	GDS4094	E2F1	KD	1	1	1	1
52	GDS4094	E2F2	KD	0	1	0	0
53	GDS4095	NCOA	KD	0	0	0	0
54	GDS4280	BCOR	VR	1	1	0	0
55	GDS4294	RARA	KO	1	1	0	0
56	GDS4302	GFI1B	OE	1	0	1	0
57	GDS4309	EZH2	KD	1	1	1	0
58	GDS4315	OTT1	KO	0	0	0	0
59	GDS4315	RBM15	KO	1	0	0	0
60	GDS4320	PPARB	KO	0	0	0	0
61	GDS4320	PPARD	KO	1	1	1	0
62	GDS4341	MIST1	KD	0	0	0	0
63	GDS4348	PDX1	KD	1	1	1	0
64	GDS4370	PPARG	KD	1	1	1	0
65	GDS4373	AIRE	KO	1	1	0	0
66	GDS4386	CTNNB1	KD	1	1	1	0
67	GDS4388	SIN3A	KD	1	1	1	1
68	GDS4407	CEBPA	VR	1	1	1	0
69	GDS4416	NOD2	VR	1	1	0	0
70	GDS514	NFE2L2	KO	1	1	1	0
71	GDS791	ESR2	KO	1	1	1	0
72	GDS998	TCOF1	KD	1	1	0	0
73	GDS998	TCOF1	OE	1	1	0	0

Table S1 TF perturbation experiment details

	GEO ID	Drug	TF present on array	Interacting genes present on arrray
1	GDS1050	valproic acid	1	1
2	GDS1273	amoxicillin	0	0
3	GDS1333	oxandrolone	1	1
4	GDS1334	oxandrolone	1	1
5	GDS1617	zinc acetate	0	0
6	GDS1808	glipizide	1	1
7	GDS1808	rosiglitazone	1	1
8	GDS1842	ethanol	0	0
9	GDS1864	levetiracetam	1	1
10	GDS2021	metoprolol	1	1
11	GDS2037	isoflurane	1	1
12	GDS2107	ethanol	1	1
13	GDS2314	dexamethasone	1	1
14	GDS2324	estradiol	1	1
15	GDS2453	rosiglitazone	1	1
16	GDS2456	dactinomycin	0	0
17	GDS2494	sirolimus	1	1
18	GDS2531	clozapine	1	1
19	GDS2531	haloperidol	1	1
20	GDS253	methylprednisolone	1	1
21	GDS2605	niacin	1	1
22	GDS2608	olanzapine	1	1
23	GDS2767	ethanol	1	1
24	GDS2772	propofol	1	1
25	GDS2772	sevoflurane	1	1
26	GDS2777	bexarotene	1	1
27	GDS2802	dexamethasone	1	1
28	GDS2878	titanium dioxide	0	0
29	GDS2913	diethylstilbestrol	1	1
30	GDS2970	chlorambucil	0	0
31	GDS2983	triclosan	0	0
32	GDS2998	permethrin	1	0
33	GDS3002	valproic acid	1	1
34	GDS3012	decitabine	1	1
35	GDS3042	imatinib	1	1
36	GDS3043	imatinib	1	1
37	GDS3044	imatinib	1	1
38	GDS3045	imatinib	1	1
39	GDS3046	imatinib	1	1
40	GDS3047	imatinib	1	1
41	GDS3048	imatinib	1	1
42	GDS3049	imatinib	1	1
43	GDS3071	hydrocortisone	1	1
44	GDS3089	tretinoin	1	1
45	GDS3099	cisplatin	0	0

46	GDS3101	cisplatin	0	0
47	GDS3109	sunitinib	1	1
48	GDS3116	letrozole	1	1
49	GDS3136	triclosan	0	0
50	GDS3194	medroxyprogesterone acetate	0	0
51	GDS3215	isotretinoin	1	1
52	GDS3217	estradiol	1	1
53	GDS3218	isotretinoin	1	1
54	GDS3251	tobramycin	0	0
55	GDS3283	estradiol	1	1
56	GDS3315	estradiol	1	1
57	GDS3369	zinc sulfate	0	0
58	GDS3396	rosiglitazone	1	1
59	GDS3514	doxorubicin	1	1
60	GDS3518	imatinib	1	1
61	GDS3603	sirolimus	1	1
62	GDS3619	probucol	1	1
63	GDS3635	vitamin c	1	1
64	GDS3656	folic acid	1	0
65	GDS3703	ethanol	1	1
66	GDS3703	morphine	1	1
67	GDS3746	dexamethasone	1	1
68	GDS3751	clioquinol	0	0
69	GDS3829	rituximab	1	1
70	GDS3850	pioglitazone	1	1
71	GDS3850	rosiglitazone	1	1
72	GDS3850	troglitazone	1	1
73	GDS3946	dexamethasone	1	1
74	GDS4003	cyclophosphamide	0	0
75	GDS4004	cyclophosphamide	0	0
76	GDS4005	cyclophosphamide	0	0
77	GDS4019	pioglitazone	1	1
78	GDS4036	rosiglitazone	1	1
79	GDS4047	imatinib	1	1
80	GDS4052	estradiol	1	1
81	GDS4078	bexarotene	1	1
82	GDS4089	bortezomib	1	1
83	GDS4095	tamoxifen	1	1
84	GDS4132	pioglitazone	1	1
85	GDS4171	menadione	1	1
86	GDS4175	imatinib	1	1
87	GDS4177	imatinib	1	1
88	GDS4180	tretinoin	1	1
89	GDS4272	methotrexate	1	1
90	GDS4294	tretinoin	1	1
91	GDS4372	ursodeoxycholic acid	0	0
92	GDS4391	ribavirin	1	1
93	GDS4413	pioglitazone	1	1

94	GDS734	troglitazone	1	1
95	GDS838	imatinib	1	1
96	GDS964	methylprednisolone	1	1
97	GDS965	methylprednisolone	1	1
98	GDS972	methylprednisolone	1	1
99	GDS982	diethylstilbestrol	1	1
100	GSE10433	isotretinoin	1	1
101	GSE1063	vitamin e	0	0
102	GSE11343	rosiglitazone	1	1
103	GSE11352	estradiol	1	1
104	GSE11440	methotrexate	1	1
105	GSE11919	vitamin c	1	1
106	GSE12972	doxorubicin	1	1
107	GSE16683	estradiol	1	1
108	GSE1839	estradiol	1	1
109	GSE19136	paclitaxel	1	1
110	GSE21266	ursodeoxycholic acid	0	0
111	GSE21329	pioglitazone	1	1
112	GSE2251	estradiol	1	1
113	GSE2354	amoxicillin	0	0
114	GSE23725	menadione	1	1
115	GSE2547	olanzapine	1	1
116	GSE32962	prednisolone	1	1
117	GSE3311	ethanol	1	1
118	GSE33455	docetaxel	1	1
119	GSE37676	vitamin c	1	1
120	GSE4668	estradiol	1	1
121	GSE5007	tretinoin	1	1
122	GSE5462	letrozole	1	1
123	GSE6206	cisplatin	0	0
124	GSE6410	cisplatin	0	0
125	GSE6467	clozapine	1	1
126	GSE6511	clozapine	1	1
127	GSE6914	gemcitabine	1	1
128	GSE7114	cyclophosphamide	0	0
129	GSE9166	trovafloxacin	0	0
130	GSE9412	methotrexate	1	1

Table S2. Drug perturbation experiment details (S-2)

	Ch.Dir	t test	SAM	Fold Change
Ch.Dir	1	0.320031	0.949913	0.950498
t test	0.320031	1	0.720549	0.437619
SAM	0.949913	0.720549	1	0.721472
Fold Change	0.950498	0.437619	0.721472	1

Table S3. TF perturbations: TF rankings CDF

	Ch.Dir	t test	SAM	Fold Change
Ch.Dir	1	0.001237	0.014379	7.98E-10
t test	0.001237	1	0.89801	0.004785
SAM	0.014379	0.89801	1	0.000929
Fold Change	7.98E-10	0.004785	0.000929	1

Table S4. TF perturbations: Interactor rankings CDF

	Ch.Dir	t test	SAM	Fold Change
Ch.Dir	1	0	0	0
t test	0	1	0.1333	0
SAM	0	0.1333	1	0
Fold Change	0	0	0	1

Table S5. TF perturbations: ChEA binding site associated gene rankings CDF

	Ch.Dir	t test	SAM	Fold Change
Ch.Dir	1	0	0	0
t test	0	1	0.86352	2.77E-10
SAM	0	0.86352	1	2E-12
Fold Change	0	2.77E-10	2E-12	1

Table S6. TF perturbations: Encode binding site associated gene rankings CDF

	Ch.Dir	t test	SAM	Fold Change
Ch.Dir	1	0.179765	0.130491	0.013079
t test	0.179765	1	0.999625	0.050509
SAM	0.130491	0.999625	1	0.041362
Fold Change	0.013079	0.050509	0.041362	1

Table S7. Drug perturbations: Drug target rankings CDF

	Ch.Dir	t test	SAM	Fold Change
Ch.Dir	1	6.48E-11	1.19E-09	0
t test	6.48E-11	1	0.794584	1.31E-08
SAM	1.19E-09	0.794584	1	1.63E-08
Fold Change	0	1.31E-08	1.63E-08	1

Table S8. Drug perturbations: Drug target interactor rankings CDF

References

1. Hastie T, Tibshirani R, Friedman JJH: **The elements of statistical learning**, vol. 1: Springer New York; 2001.
2. Guo Y, Hastie T, Tibshirani R: **Regularized linear discriminant analysis and its application in microarrays**. *Biostatistics* 2007, **8**(1):86-100.
3. Hastie T, Tibshirani R: **Efficient quadratic regularization for expression arrays**. *Biostatistics* 2004, **5**(3):329-340.
4. Ye J, Xiong T, Li Q, Janardan R, Bi J, Cherkassky V, Kambhamettu C: **Efficient model selection for regularized linear discriminant analysis**. In: *Proceedings of the 15th ACM international conference on Information and knowledge management: 2006*. ACM: 532-539.
5. Risvik H: **Principal component analysis (PCA) & NIPALS algorithm**. In.; 2007.
6. Knyazev AV, Argentati ME: **Principal angles between subspaces in an A-based scalar product: algorithms and perturbation estimates**. *SIAM Journal on Scientific Computing* 2002, **23**(6):2008-2040.
7. Budhraja V, Spitznagel E, Schaiff WT, Sadovsky Y: **Incorporation of gene-specific variability improves expression analysis using high-density DNA microarrays**. *BMC biology* 2003, **1**(1):1.
8. Hsiao A, Worrall D, Olefsky J, Subramaniam S: **Variance-modeled posterior inference of microarray data: detecting gene-expression changes in 3T3-L1 adipocytes**. *Bioinformatics* 2004, **20**(17):3108-3127.
9. Tusher VG, Tibshirani R, Chu G: **Significance analysis of microarrays applied to the ionizing radiation response**. *Proceedings of the National Academy of Sciences* 2001, **98**(9):5116-5121.
10. Smyth GK: **Linear models and empirical bayes methods for assessing differential expression in microarray experiments**. *Stat Appl Genet Mol Biol* 2004, **3**(1):3.
